# Young Male with Seizures

**DOI:** 10.5811/cpcem.2018.10.40840

**Published:** 2019-01-04

**Authors:** S. Manu Ayyan, Binoy X. Kaliparrambil, Suresh G. Nair

**Affiliations:** Academy of Medical Sciences, Department of Emergency Medicine, Pariyaram, Kerala, India

## CASE PRESENTATION

**Image 1:**
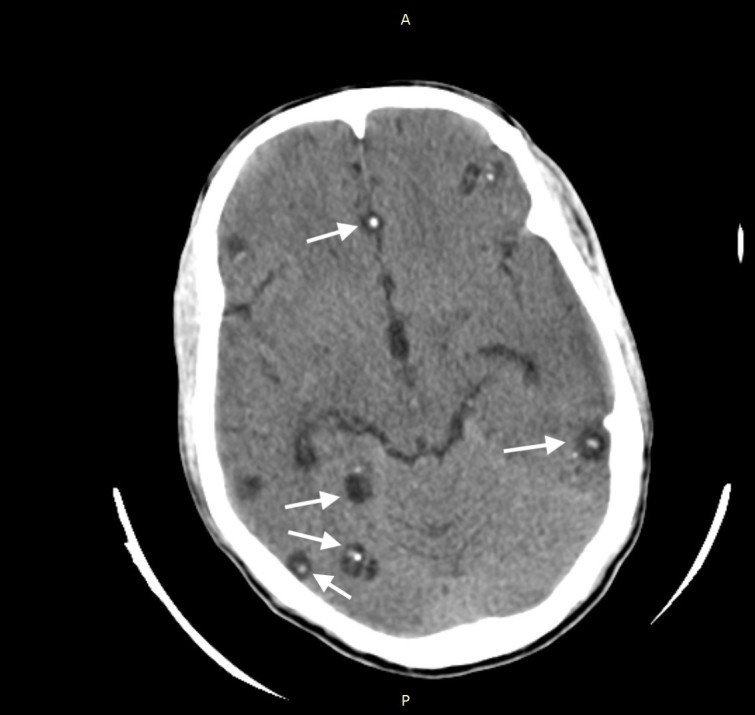
Computed tomography of the brain showing multiple cystic lesions and cyst with dot sign in patient with neurocystocercosis (arrows).

**Image 2:**
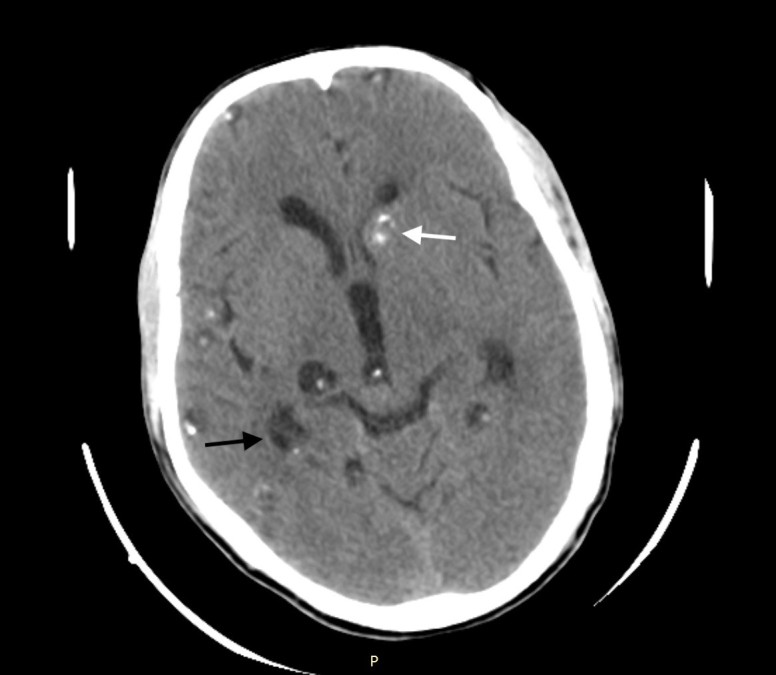
Computed tomography of the brain showing multiple cystic lesions in different stages: vesicular (black arrow) and nodular calcific stage with lesion in left cerebral hemisphere near basal ganglion showing calcification (white arrow).

**Image 3:**
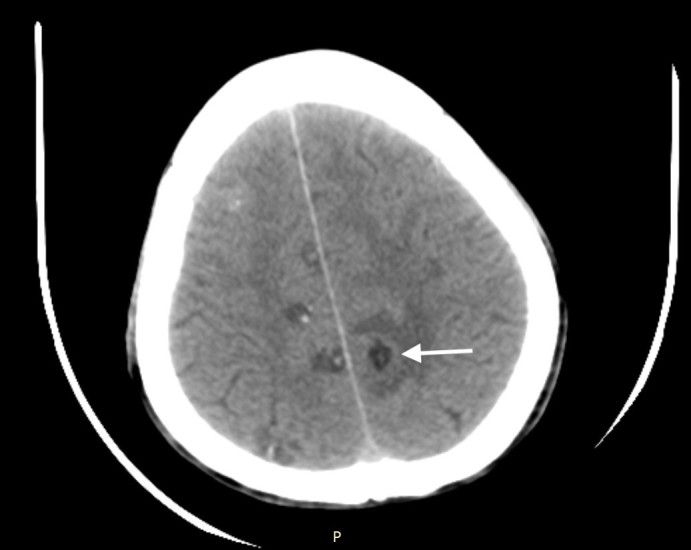
Computed tomography of the brain showing multiple cystic lesions in vesicular and colloid-vesicular stage with lesion in left cerebral hemisphere demonstrating surrounding edema (arrow).

A 30-year-old Asian male presented with a history of generalized tonic-clonic seizures an hour before presenting to emergency department. He had a similar episode three years prior for which he had not sought any medical evaluation. He was conscious and oriented on presentation, and physical examination was unremarkable. Non-contrast computed tomography (CT) of the head revealed multiple cystic lesions on both cerebral hemispheres in different stages (Images 1–3).

## DIAGNOSIS

Neurocysticercosis (NCC), is the most common parasitic disease of the central nervous system and is caused by the larval form (cysticercus) of the tapeworm *Taenia solium*. It is the most common cause of acquired epilepsy worldwide. Seizures are the most common manifestation, present in 70–90% of symptomatic patients.^1^ NCC has primarily been a disease that remains endemic in less economically developed countries; however, because of globalization, NCC is now being diagnosed more frequently in high-income countries.^2^

A set of diagnostic criteria for NCC has been proposed and revisited. These criteria are useful for maintaining uniformity, particularly for research.^3^,^4^ Absolute criteria include direct visualization of parasite, histological demonstration of parasite or evidence of cystic lesion with scolex on CT or magnetic resonance imaging. The disease can be parenchymal, occurring in the brain substance, or extraparenchymal, occurring in the ventricles, basilar cisterns, or subarachnoid space of the brain or in the spinal cord. The scolex is visualized as a bright, extramural nodule within the cyst (hole with dot appearance). Parenchymal NCC has four stages: vesicular; colloidal vesicular; granular nodular; and nodular calcified. When multiple cysts in different stages of evolution are visible it gives rise to the “starry-sky” appearance, which is typical of NCC.

Emergency therapeutic interventions are aimed at managing the neurological complications, which include anticonvulsant therapy, corticosteroids, neurosurgical intervention and/or treatment of increased intracranial pressure. Cysticidal therapy is indicated with antihelminthics (albendazole or praziquantel), but must be administered with caution because larval death provokes an inflammatory response that may increase symptoms. Concomitant steroids are usually indicated. Treatment with cysticidal therapy leads to reduction in seizure frequency and a faster resolution of lesions.
